# Overcoming chemoresistance in nasopharyngeal carcinoma: minocycline as a mitochondrial translation inhibitor

**DOI:** 10.1007/s12672-025-03598-6

**Published:** 2026-01-27

**Authors:** Fang Xiang, Qiong Dai, Wei Chen

**Affiliations:** 1 Department of Otolaryngology, The Central Hospital of Wuhan, Tongji Medical College, Huazhong University of Science and Technology, Wuhan, China; 2 State Key Laboratory of Ultrasound in Medicine and Engineering, Chongqing Medical University, Chongqing, China

**Keywords:** Mitochondrial translation, EF-Tu, Minocycline, Nasopharyngeal carcinoma, Chemoresistance

## Abstract

Chemo-resistance poses a major challenge in nasopharyngeal carcinoma (NPC) treatment, necessitating novel therapeutic approaches. Through a chemical screen, we identified minocycline as a selective inhibitor of chemo-resistant NPC cells, demonstrating potent cytotoxicity while sparing non-cancerous cells. Mechanistically, minocycline inhibited mitochondrial translation, leading to reduced activities of mitochondrial complexes I and IV, impaired oxygen consumption, and disrupted mitochondrial respiration. Its cytotoxic effects were dependent on oxygen availability and an intact mitochondrial respiratory chain, as evidenced by its diminished efficacy under anoxic conditions. Genetic knockdown of mitochondrial elongation factor Tu (EF-Tu), a critical regulator of mitochondrial translation, mimicked the effects of minocycline, further validating mitochondrial translation as a therapeutic target. In a chemo-resistant NPC xenograft model, minocycline significantly suppressed tumor growth, reduced Ki-67 expression, and impaired mitochondrial function in tumor-derived cells. These findings highlight mitochondrial translation inhibition as a promising strategy to overcome chemo-resistance in NPC and identify minocycline as a potential therapeutic agent.

## Introduction

Nasopharyngeal carcinoma (NPC) is a malignant epithelial tumor originating in the nasopharyngeal mucosa, with a high incidence in Southeast Asia and Southern China [1]. Its etiology is multifactorial, involving a combination of Epstein–Barr virus (EBV) infection, genetic susceptibility, and environmental factors such as consumption of salted or preserved foods, tobacco smoking, and occupational exposure to wood dust or formaldehyde [2]. EBV infection plays a critical role in NPC development, contributing to tumorigenesis through the expression of latent viral proteins that promote cell proliferation, immune evasion, and genomic instability [3]. Despite advances in chemoradiotherapy, which remains the standard of care for NPC, recurrent and metastatic disease frequently develops due to treatment resistance [4]. Mechanisms underlying resistance include enhanced DNA repair, tumor microenvironment, epithelial-to-mesenchymal transition (EMT), and metabolic reprogramming [5–7]. Notably, chemo-resistant cancer cells often shift toward increased reliance on mitochondrial oxidative phosphorylation (OXPHOS) for energy production, which supports their survival and growth under therapeutic stress [5].

Unlike the conventional view of tumor metabolism dominated by glycolysis (the Warburg effect), many tumor cells, particularly chemo-resistant and metastatic cancer cells, exhibit increased dependence on mitochondrial OXPHOS [8–10]. This metabolic reprogramming enables cancer cells to meet their bioenergetic and biosynthetic demands, particularly under conditions such as drug treatment. This reliance on OXPHOS is a distinguishing feature of chemo-resistant cancer cells compared to their sensitive counterparts and normal cells, making mitochondrial function an attractive therapeutic target [10]. Among mitochondrial processes, mitochondrial translation is essential for the synthesis of key components of the electron transport chain (ETC) that drives OXPHOS. There are the 13 mitochondrial DNA (mt-DNA)-encoded proteins are translated by mitochondrial ribosomes within the mitochondrial matrix [11]. Mitochondrial elongation factor Tu (EF-Tu) is a critical regulator of this process, facilitating the delivery of aminoacyl-tRNAs to the decoding site on the mitochondrial ribosome [12]. EF-Tu inhibition disrupts mitochondrial translation impairing ETC function and reducing ATP production.

Minocycline is a second-generation tetracycline antibiotic that has been widely used for its antimicrobial and anti-inflammatory properties [13]. It inhibits bacterial protein synthesis by binding to the 30 S ribosomal subunit and blocking aminoacyl-tRNA attachment, thereby preventing peptide chain elongation [14]. In recent years, minocycline has gained attention for its pleiotropic effects, including neuroprotective and anti-cancer activities. Several preclinical studies have demonstrated that minocycline can induce apoptosis, inhibit cell migration and invasion, and enhance chemosensitivity in various cancer models [15–17].

In this study, we screened a library of drugs, focusing on those known to target mitochondria, for their ability to inhibit the growth and viability of chemo-resistant NPC cells while sparing normal counterparts. Based on the results of this screen, we further investigated the impact of genetic inhibition of mitochondrial translation factors on mitochondrial function and chemo-resistant NPC cell growth and viability. These findings provide a foundation for developing mitochondrial translation inhibitors as a novel approach to overcome NPC chemo-resistance.

## Materials and methods

### Cell culture and treatment conditions

NPC cell lines were obtained from Cell Bank of Type Culture Collection of the Chinese Academy of Sciences and were grown in RPMI-1640 (Gibco) supplemented with 10% fetal bovine serum (FBS) and 1% penicillin-streptomycin. Human nasopharyngeal epithelial cells (HNEC) were obtained from PromoCell and were growth in manufacture recommended medium. CNE2-r and C666-1-r were kind gifts from Dr. Liu’s laboratory. All cell were maintained at 37 °C in a humidified atmosphere with 5% CO₂ and 20% O_2_. Notably, C666-1-r cells were seeded onto flasks or plates pre-coated with 50 µg/mL collagen I to enhance cell attachment. Hypoxic conditions (5% and 1% O₂) were achieved using a hypoxia chamber (BioSpherix, USA) set to the desired oxygen concentration. For anoxic conditions, cells were incubated in a pre-equilibrated anoxic chamber with 0% O₂ and a gas mixture of 95% N₂ and 5% CO₂. Cells were preconditioned under these oxygen levels for 12 h, followed by treatment with control or minocycline for 72 h.

### Growth and viability assay

Cell growth and viability were measured using the MTS assay. Cells were seeded in 96-well plates at a density of 8000 cells/well and treated with the indicated drug concentrations for 72 h. Following treatment, 20 µL of MTS reagent (CellTiter 96^®^ AQueous One Solution, Promega) was added to each well, and the plate was incubated for 2 h. Absorbance at 490 nm was measured using a microplate reader.

### Small interfering RNA (siRNA) transfection

NPC cells were transfected with siRNA targeting EF-Tu or scrambled control siRNA (final concentration: 50 nM) using Lipofectamine RNAiMAX reagent (Thermo Fisher Scientific). Briefly, cells were seeded in 6-well plates and transfected with siRNA-Lipofectamine complexes prepared in Opti-MEM medium (Thermo Fisher Scientific). After 24 h, transfection efficiency was confirmed by quantitative PCR. Cells were treated or harvested for downstream assays 48 h post-transfection.

### Mitochondrial translation assay

Mitochondria were isolated from cells using the Mitochondrial Isolation Kit for Cultured Cells (Thermo Fisher Scientific) following the manufacturer’s instructions. Isolates were incubated with buffer control, minocycline for 5 min at 30 °C, followed by the addition of [³H]-leucine. Incorporation of [³H]-leucine into newly synthesized mitochondrial proteins was measured after 60 min, and radioactivity was quantified as counts per minute (Cpm) per milligram of protein using a liquid scintillation counter.

### Oxygen consumption rate (OCR) measurement

OCR was determined using a Clark-type oxygen electrode (Hansatech Instruments). Cells were harvested and resuspended in respiration buffer containing (125 mM sucrose, 65 mM KCl, 10 mM HEPES, 2 mM MgCl₂, and 2.5 mM KH₂PO₄, pH 7.4). Approximately 1 × 10⁶ cells were added to the electrode chamber maintained at 37 °C with constant stirring. Baseline OCR was recorded for 20 min, followed by the addition of oligomycin (1 µM) to inhibit ATP synthase and measure proton leak-dependent respiration. OCR was calculated as the rate of oxygen depletion (nmol O₂/min) and normalized to protein content.

### Mitochondrial complex activity assay

The activities of mitochondrial complexes I, II, and IV were measured using commercial assay kits according to the manufacturer’s instructions (Abcam). Complex I activity was determined by monitoring the oxidation of NADH at 340 nm. Complex II activity was measured by assessing the reduction of 2,6-dichlorophenolindophenol (DCPIP) at 600 nm. Complex IV activity was evaluated by monitoring the oxidation of reduced cytochrome c at 550 nm. Reactions were conducted in a spectrophotometer, and the absorbance changes over time were recorded. Activities were normalized to protein content and expressed as nmol/min/mg protein.

### Real time PCR

Total RNA was extracted from CNE2-r and C666-1-r cells using the RNeasy Mini Kit (Qiagen) according to the manufacturer’s instructions. RNA quantity and purity were assessed with a NanoDrop spectrophotometer (Thermo Fisher Scientific). One microgram of total RNA was reverse-transcribed using the High-Capacity cDNA Reverse Transcription Kit (Applied Biosystems). Quantitative real-time PCR was performed using PowerUp SYBR Green Master Mix (Applied Biosystems) and predesigned primer sets for CYC1, CYCS, IDH2, MCU, and GAPDH (Thermo Fisher Scientific) on a QuantStudio 5 Real-Time PCR System. Relative gene expression was calculated using the ΔΔCt method and normalized to GAPDH. All reactions were run in triplicate.

### In vivo tumor growth assay

A xenograft model was established using male NOD-SCID mice, with minimal influence of sex on the study results. C666-1-r cells (5 × 10⁶ cells) were resuspended in 100 µL of a 1:1 mixture of PBS and Matrigel (Corning) and subcutaneously injected into the flanks of mice. When tumors reached approximately 150 mm³, mice were randomly divided into control and treatment groups (*n* = 5 per group). Minocycline was administered via intraperitoneal injection at a dose of 50 mg/kg daily. Tumor size was measured every 5 days using digital calipers, and volume was calculated as tumor length x width^2^ × 0.5236). On day 20, mice were euthanized. Mice were excised and minced into ~ 1 mm³ fragments and digested in an enzyme solution (1 mg/mL collagenase IV and 0.1 mg/mL DNase I) at 37 °C for 1 h. The digested tissue was passed through a 70 μm cell strainer to obtain a single-cell suspension, centrifuged at 300 × g for 5 min, washed with PBS, and resuspended in complete culture medium for downstream analyses.

### Statistical analyses

All data are presented as mean ± standard derivation (SD) from at least three independent experiments. Statistical comparisons were performed using ANOVA with Tukey’s HSD using GraphPad Prism, with *p* < 0.05 considered statistically significant.

## Results

### Chemo-resistant NPC cells display higher mitochondrial function and mitochondrial gene expression than parental cells

**Fig. 1:**
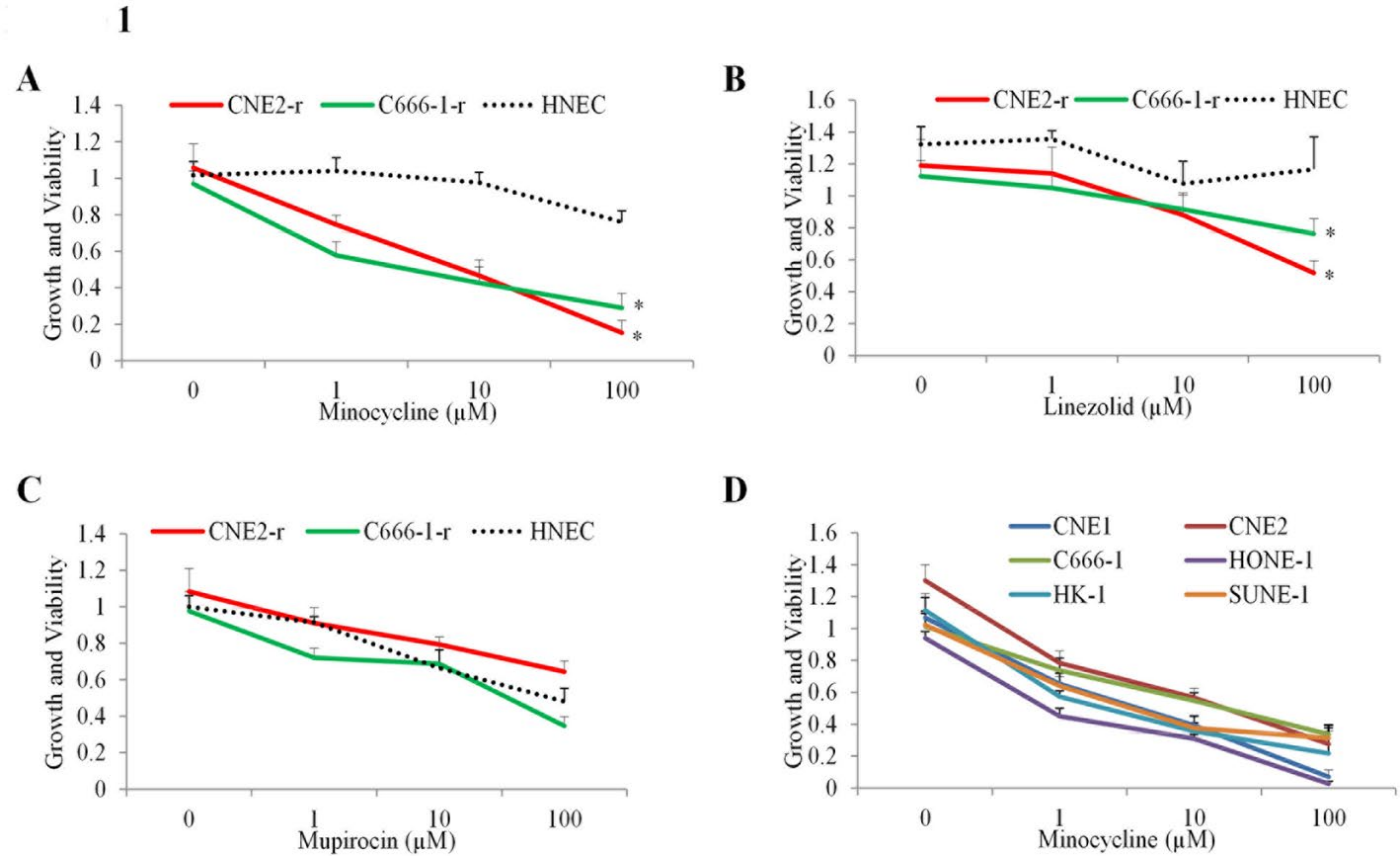
Chemical screen for compounds selectively targeting chemo-resistant NPC cells. Growth and viability of CNE2-r, C666-1-r, and HNEC (human nasopharyngeal epithelial cells) upon treatment with minocycline (A), linezolid (B) and mupirocin (C) at varying concentrations (0-100 µM). (D) Growth and viability of multiple NPC cell lines (CNE1, CNE2, C666-1, HONE-1, HK-1, and SUNE-1) treated with minocycline. Data are presented as mean ± SD (*n* = 3). Growth and viability were determined by MTS. * *P* < 0.05, compared to normal control

Given the fact that chemo-resistant NPC cells largely depend on mitochondrial function for energy production [5], we firstly compiled a library of 49 drugs known to target mitochondria with well-characterized pharmacokinetics and toxicology, and wide therapeutic windows. We then screened this library to identify agents that decreased growth and viability of C666-1-r and CNE2-r cells, that display resistance to chemotherapeutic drugs cisplatin and 5-FU [18], while sparing normal counterpart cells HNEC. Among the screened compounds, we identified three compounds that either did not have any or little previously anticancer activity but displayed some inhibitory effects on chemo-resistant NPC cells. Minocycline significantly reduced cancer cell viability across both tested cancer cell groups, with minimal effects on normal melanocytes (dotted line), indicating selective cytotoxicity (*p* < 0.01, Fig. 1A); linezolid led to a significant reduction in viability at the highest dose in one cancer cell group compared to normal cells (*p* < 0.05, Fig. 1B); however, the effect was less consistent across other doses (*p* > 0.05, Fig. 1B), making its selectivity inconclusive. In Fig. 1C, mupirocin showed no statistically significant reduction in viability across all three cell types at any dose (*p* > 0.05). Minocycline demonstrated the highest selective cytotoxicity against chemo-resistant NPC cells, with minimal effects on HNEC, which we chose to analyze further.

To evaluate the broader applicability of minocycline, its cytotoxicity was further assessed in a panel of NPC cell lines with diverse characteristics, including EBV-positive undifferentiated (C666-1), well-differentiated (CNE1), poorly differentiated (CNE2, HK-1 and HONE1) and poorly differentiated with high metastatic potential (SUNE-1). Minocycline significantly exhibited potent cytotoxic effects across all tested NPC cell lines, with IC₅₀ values at low micromolar range (Fig. 1D, *p* < 0.05). These findings highlight minocycline’s potential as a broad-spectrum therapeutic agent for NPC, particularly in the context of chemoresistance.

### Inhibition of mitochondrial translation underpins minocycline-induced growth and viability reduction in chemo-resistant NPC cells

**Fig. 2:**
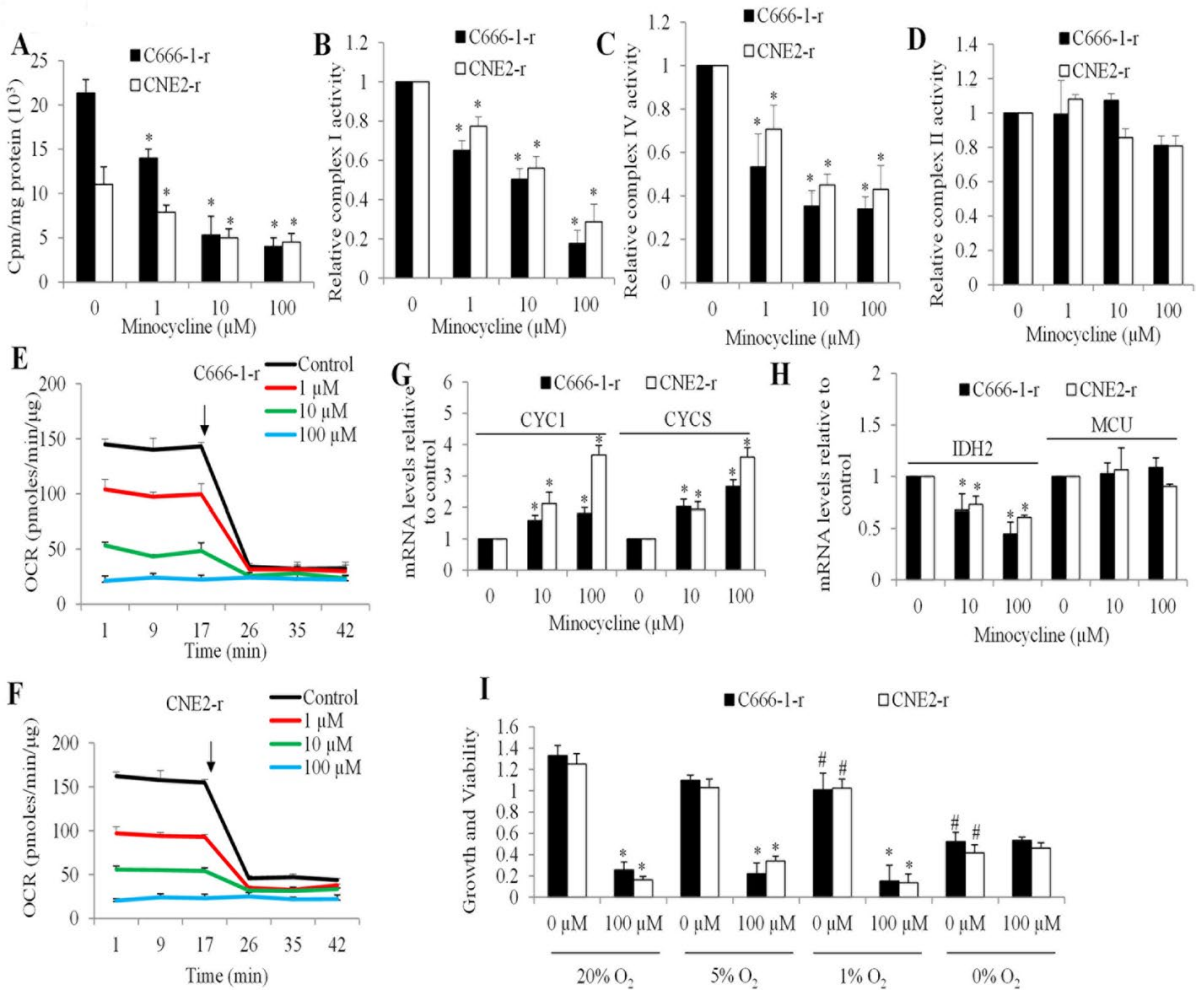
Inhibition of mitochondrial translation is functionally important for minocycline-induced inhibition of growth and survival of chemo-resistant NPC cells. (A) Mitochondrial translation assay showing incorporation of [³H]-leucine into mitochondrial isolates from CNE2-r and C666-1-r cells treated with buffer control, minocycline (1, 10, 100 µM) for 5 min at 30 °C, followed by the addition of [³H]-leucine. Incorporation of [³H]-leucine was measured after 60 min. (B-D) Relative activities of mitochondrial complexes I, II, and IV in CNE2-r and C666-1-r cells treated with minocycline. Oxygen consumption rate (OCR) of C666-1-r (E) and CNE2-r (F) cells treated with control or minocycline. The arrow denotes addition of 1 µM oligomycin. (G) Minocycline significantly increased mRNA levels of CYC1 and CYCS. (H) Minocycline significantly decreased mRNA levels of IDH2 without affecting MCU. (I) Growth and viability of C666-1-r and CNE2-r cells treated with control or 100 µM minocycline under various oxygen conditions (20%, 5%, 1%, and 0% O₂). Data are presented as mean ± SD (*n* = 3). * *P* < 0.05, compared to control (0 µM); # *P* < 0.05, compared to 20% O_2_

Minocycline is known to target bacterial mitochondrial ribosomes, interfering with mitochondrial translation [19]. To investigate whether its selective toxicity against chemo-resistant NPC cells is mediated through a similar mechanism, we performed cell-free mitochondrial translation assay using mitochondria isolated from chemo-resistant cells. Minocycline significantly reduced the incorporation of [³H]-leucine into newly synthesized mitochondrial proteins in CNE2-r and C666-1-r (Fig. 2A), demonstrating its inhibitory effect on mitochondrial translation. Since respiratory complexes I and IV require mitochondrially translated proteins in their assembly, while complex II does not [20], we examined the activities of these complexes following minocycline treatment. Minocycline caused a dose-dependent reduction in the activities of complexes I and IV (Fig. 2B and C), whereas the enzymatic activity of complex II was less affected (Fig. 2D). These results indicate that minocycline selectively disrupts mitochondrial respiratory chain components dependent on mitochondrial translation. Consistent with these findings, minocycline treatment led to a significant decrease in oxygen consumption rates (OCR) in CNE2-r and C666-1-r cells, further confirming its impact on mitochondrial respiration (Fig. 2E and F).

To further explore the impact of minocycline on mitochondrial function, we analysed key genes involved in oxidative phosphorylation and the tricarboxylic acid cycle. In both CNE2-r and C666-1-r cells, minocycline treatment significantly upregulated CYC1 and CYCS mRNA levels (Fig. 2G), consistent with a compensatory response to impaired mitochondrial respiration. Expression of IDH2, a mitochondrial TCA cycle enzyme, was modestly downregulated (Fig. 2H), suggesting reduced metabolic flux through the TCA cycle. In contrast, MCU expression remained largely unchanged (Fig. 2H). These findings further support that minocycline induces mitochondrial stress and disrupts bioenergetic homeostasis in chemo-resistant NPC cells.

To assess whether minocycline’s effects on the growth and viability of NPC cells depend on mitochondrial function, we evaluated its activity under varying oxygen conditions, ranging from hypoxia (5% and 1% O₂) to anoxia (0% O₂). Hypoxic and anoxic conditions alone significantly reduced the viability of CNE2-r and C666-1-r cells (Fig. 2I). Notably, under anoxic conditions, co-treatment with minocycline did not cause additional reductions in cell growth or viability (Fig. 2I). Interestingly, we also observed significant reduction of cell growth under hypoxic/anoxic condition (1% and 0% O_2_) compared to normoxia (20% O_2_) (Fig. 2I). These findings suggest that minocycline’s cytotoxic effects require oxygen availability and an intact mitochondrial respiratory chain. Together, these results demonstrate that the anti-tumor activity of minocycline in chemo-resistant NPC cells is mediated by its inhibition of mitochondrial translation and subsequent disruption of mitochondrial respiration, which is dependent on oxygen availability.

### Genetic Inhibition of mitochondrial translation is effective against chemo-resistant NPC cells

**Fig. 3:**
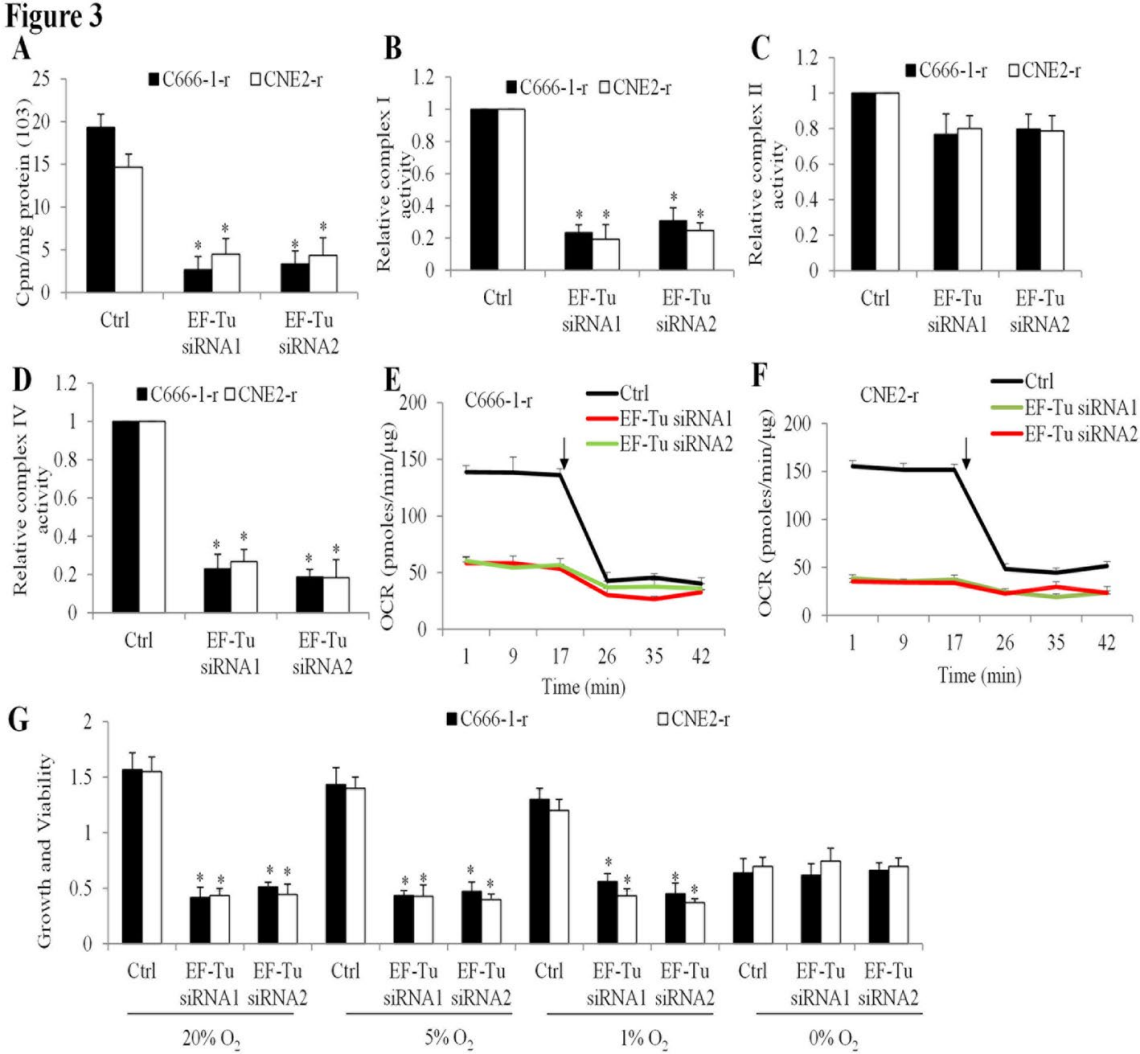
EF-Tu knockdown suppresses mitochondrial function and cell viability in chemo-resistant NPC cells. (A) Mitochondrial translation assay showing incorporation of [³H]-leucine into mitochondrial isolates from CNE2-r and C666-1-r cells transfected with control or EF-Tu siRNA (siRNA1 and siRNA2). (B-D) Relative activities of mitochondrial complexes I, II, and IV in CNE2-r and C666-1-r cells transfected with control or EF-Tu siRNA. OCR of C666-1-r (E) and CNE2-r (F) cells transfected with control or EF-Tu siRNA over time. The arrow denotes addition of 1 µM oligomycin. (G) Growth and viability of C666-1-r and CNE2-r cells transfected with control or EF-Tu siRNA under various oxygen conditions (20%, 5%, 1%, and 0% O₂). Data are presented as mean ± SD (*n* = 3)

To further investigate the therapeutic potential of mitochondrial translation inhibition in chemo-resistant NPC cells, we targeted mitochondrial elongation factor Tu (EF-Tu), a key protein that facilitates the delivery of aminoacyl-tRNAs to the mitochondrial ribosome’s decoding site [21]. EF-Tu expression was silenced in CNE2-r and C666-1-r cells using siRNA, and its effects were evaluated using the same assays performed for minocycline. EF-Tu knockdown significantly impaired mitochondrial translation (Fig. 3A) and reduced the activities of mitochondrial complexes I and IV, while having minimal impact on complex II activity (Figs. 3B-D). Mitochondrial respiration was also markedly decreased in EF-Tu-silenced cells (Fig. 3E and F). Importantly, EF-Tu knockdown led to a significant reduction in cell viability, mirroring the cytotoxic effects observed with minocycline (Fig. 3G). Notably, under anoxic conditions, EF-Tu knockdown did not cause further reductions in cell growth or viability (Fig. 3G). The phenotypic similarity between EF-Tu knockdown and minocycline treatment supports the notion that targeting mitochondrial translation is a promising therapeutic strategy for chemo-resistant NPC.

### Minocycline inhibits tumor growth and mitochondrial function in xenograft model of chemo-resistant NPC

**Fig. 4:**
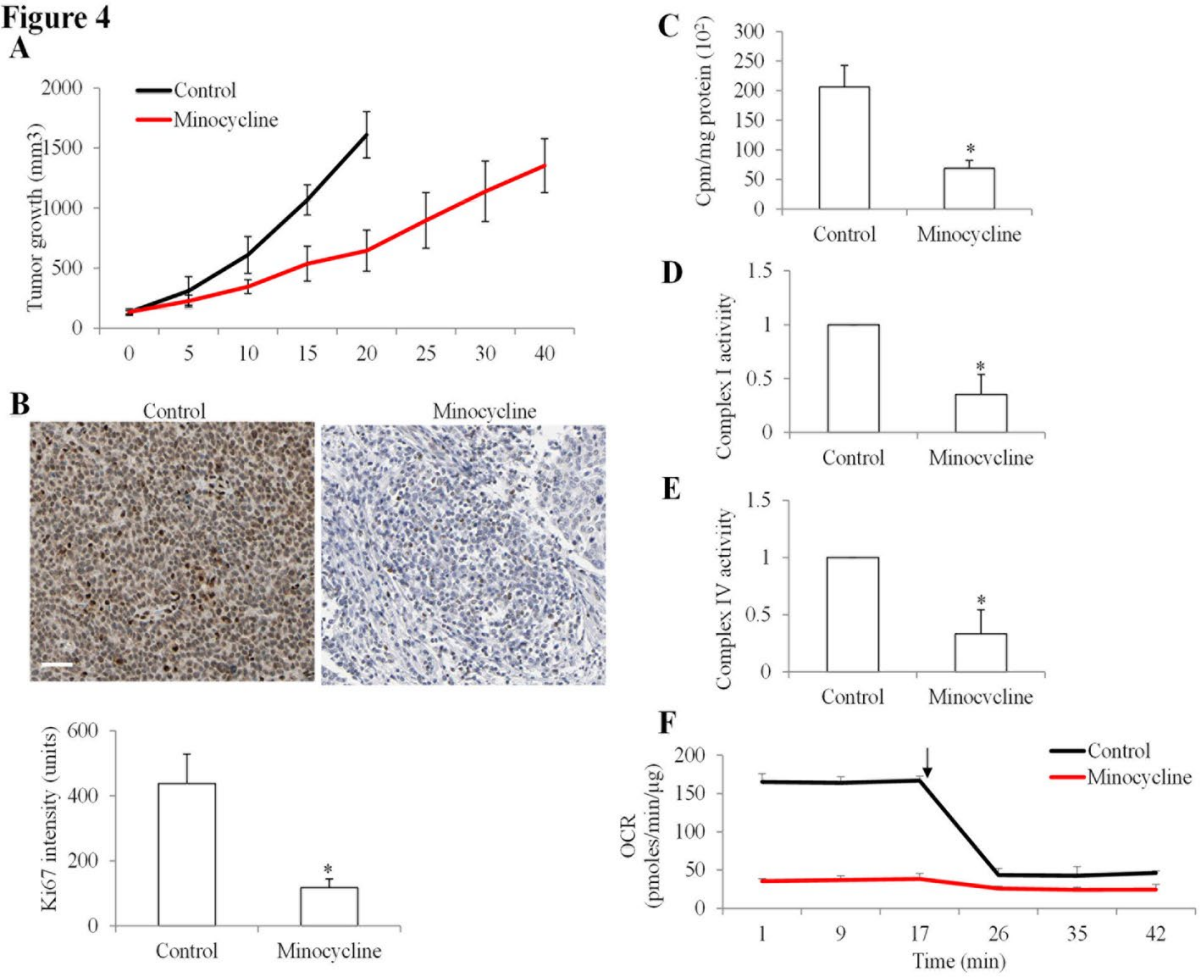
Minocycline inhibits chemo-resistant NPC cell growth and mitochondrial functions in vivo. (A) Tumor growth in an in vivo C666-1-r xenograft model treated with control or minocycline. Tumor volume was measured at indicated time points. Data are presented as mean ± SD (*n* = 5). One-way repeated measures ANOVA revealed a significant main effect of treatment (*p* < 0.01). (B) Representative immunohistochemistry images showing Ki-67 staining in tumors treated with control or minocycline. Quantification of Ki-67 intensity is shown below. Scale bar represents 50 μm. Magnification 200x. (C) Mitochondrial translation activity of cells isolated from tumors treated with control or minocycline. (D, E) Activities of mitochondrial complexes I and IV in cells isolated from tumors treated with control or minocycline. (F) OCR of cells isolated from tumors treated with control or minocycline over time. The arrow denotes addition of 1 µM oligomycin. Data are presented as mean ± SD (*n* = 3). *, *P* < 0.05 compared to control

To assess the anti-tumor efficacy of minocycline in vivo, we established a chemo-resistant NPC xenograft model by subcutaneously injecting C666-1-r cells into immunodeficient mice. When the tumors reached an average volume of approximately 150 mm³, the mice were randomized into control and treatment groups. The treatment group received minocycline (50 mg/kg) via daily intraperitoneal injections, while the control group received the vehicle alone. Minocycline treatment significantly slowed tumor growth compared to the control group (Fig. 4A). By day 20, the average tumor volume in the minocycline-treated group was reduced by 50% relative to controls. Immunohistochemical analysis further revealed a marked reduction in Ki-67 expression in minocycline-treated tumors, indicating decreased tumor cell proliferation (Fig. 4B).

To explore the mechanisms underlying these effects, tumor cells were isolated from control and minocycline-treated mice, and their mitochondrial function was assessed. Mitochondrial translation assays demonstrated significantly reduced incorporation of [³H]-leucine into newly synthesized mitochondrial proteins in cells derived from minocycline-treated tumors (Fig. 4C). Similarly, the activities of mitochondrial complexes I and IV were markedly diminished in minocycline-treated tumors (Fig. 4D and E). Additionally, oxygen consumption rate (OCR) measurements revealed impaired mitochondrial respiration in cells from minocycline-treated tumors compared to controls (Fig. 4F). Taken together, these results demonstrate that minocycline suppresses chemo-resistant NPC tumor growth in vivo by inhibiting mitochondrial translation, impairing mitochondrial function, and reducing tumor cell proliferation.

## Discussion

This study highlights the potential of targeting mitochondrial translation as a therapeutic strategy for overcoming chemo-resistance in NPC. Leveraging previous findings that chemo-resistant cancer cells, including NPC, rely heavily on mitochondrial OXPHOS for energy production and survival under therapeutic stress [5, 22], we identified minocycline as a potent inhibitor of chemo-resistant NPC cell growth and viability. Minocycline exerts its anti-tumor effects by targeting mitochondrial translation, leading to impaired respiratory chain function and decreased energy production. Our findings further validate mitochondrial translation as a critical vulnerability in chemo-resistant cancer cells and add NPC to the growing list of cancers targeted by minocycline.

Chemo-resistance in NPC remains a significant clinical challenge, resulting in poor prognosis for patients with recurrent or metastatic disease. Prior studies have reported that chemo-resistant cancer cells often reprogram their metabolism to depend more on OXPHOS compared to their parental counterparts [9, 23, 24]. Consistent with these reports, our findings demonstrate that minocycline disrupts mitochondrial translation, impairing OXPHOS-dependent processes such as respiratory complex activities and oxygen consumption, which are critical for tumor cell survival.

Minocycline has demonstrated anti-cancer activities in both pre-clinical and clinical studies. Pre-clinically, minocycline binds and inhibits LYN kinase, preventing STAT3-mediated metastasis in colorectal cancer, and induces apoptosis while suppressing matrix metalloproteinase expression in breast cancer cells [15, 25]. Additionally, minocycline synergizes with cisplatin to induce S phase arrest and apoptosis in hepatocellular carcinoma cells and enhances chemotherapy efficacy in pancreatic ductal adenocarcinoma when combined with photodynamic priming [17, 26]. Clinical studies have also shown that minocycline improves outcomes in EGFR-mutant non-small cell lung cancer patients treated with EGFR-TKIs [16].

Moreover, a recent clinical trial demonstrated the feasibility and tolerability of minocycline for reducing patient-reported symptom severity during radiation therapy for head and neck cancer [27]. The study found that minocycline was effective in alleviating systemic symptoms, highlighting its safety profile and additional benefits during cancer treatment. These findings underscore the potential for repurposing minocycline in NPC therapy. The demonstrated tolerability and symptom-relieving properties of minocycline could offer a dual advantage in NPC treatment by both directly targeting chemo-resistant tumor cells and improving the quality of life for patients undergoing therapy.

Our study extends these findings by demonstrating minocycline’s selective cytotoxicity against chemo-resistant NPC cells through a novel mechanism involving mitochondrial translation inhibition. Minocycline is known to bind to the mitochondrial ribosome and directly interfere with mitochondrial protein synthesis [19]. We demonstrated that minocycline significantly reduced the incorporation of [³H]-leucine into newly synthesized mitochondrial proteins, indicating suppression of mitochondrial translation. It is less likely that minocycline suppresses the expression of EF-Tu as EF-Tu is not a mitochondrially translated protein [28]. Rather, minocycline likely exerts its effects by functionally impairing the mitochondrial ribosome itself. Importantly, we are the first to report the ability of minocycline to target chemo-resistant cancer cells specifically, addressing a critical unmet need in NPC therapy. The phenotypic similarity between EF-Tu knockdown and minocycline treatment further supports the therapeutic potential of targeting mitochondrial translation.

In vivo, minocycline significantly suppressed tumor growth in a chemo-resistant NPC xenograft model. Tumors from minocycline-treated mice exhibited decreased expression of Ki-67, indicative of reduced cellular proliferation. Furthermore, mitochondrial translation, respiratory complex activities, and oxygen consumption rates were markedly impaired in tumor-derived cells from treated mice, corroborating the in vitro findings. These results highlight the efficacy of minocycline in targeting mitochondrial function to suppress chemo-resistant NPC tumors. While our findings emphasize the therapeutic potential of minocycline, further studies are warranted to optimize dosing regimens, evaluate combination therapies, and assess its efficacy in clinical settings. Exploring the applicability of mitochondrial translation inhibitors across other cancer types with high OXPHOS dependency could further broaden the therapeutic utility of this approach.

In conclusion, our study demonstrate that inhibition of mitochondrial translation is a therapeutic strategy to overcome NPC chemoresistance. In addition, our work identified minocycline as promising candidates that could potentially enhance the treatment of NPC patients.

## Data Availability

The datasets generated and/or analysed during the current study are included in this published article. Additional data supporting the findings of this study are available from the corresponding author upon reasonable request. Deposition in a public repository is not applicable for this study.
